# Modified Wallace anastomotic technique reduces ureteroenteric stricture rates after ileal conduit urinary diversion

**DOI:** 10.1590/S1677-5538.IBJU.2019.0417

**Published:** 2020-02-20

**Authors:** Petar Kavaric, Sabovic Eldin, Radovic Nenad, Pratljacic Dragan, Marko Vukovic

**Affiliations:** 1 Clinical Center of Montenegro Department of Urology LjubljanskaPodgorica Montenegro Department of Urology, Clinical Center of Montenegro, Ljubljanska, Podgorica, Montenegro

**Keywords:** Cystectomy, Urinary Diversion, Quality of Life

## Abstract

**Purpose:**

To compare perioperative outcomes, complications and anastomotic stricture rate in a contemporary series of patients who underwent open radical cystectomy (RC) with modified Wallace anastomotic technique versus traditional ileal conduit.

**Materials and methods:**

Study enrolled 180 patients, of whom 140 were randomized and underwent RC; seventy were randomized to group I and the seventy to the group II. For the primary objective, we hypothesized that the rate of ureteroenteric strictures would be at least 20 % lower in the second group. Secondary end points included rate of anastomotic leak, surgical time, deterioration of the upper tract, intraoperative blood loss and patient-reported quality of life (HRQOL). The modified Wallace 1 technique involved eversion of the ureteral plate and bowel mucosa edges, which were anastomosed together in running fashion, while the outher anastomotic wall was augmented with sero-serosal interrupted sutures.

**Results:**

The mean (SD) follow-up time was 26.1 (5.7) months in group I and 25.2 (4.8) months in group II, during which, anastomotic stricture was observed in 8 patients (12%) from the first and 2 patients (3%) from the second group (p < 0.05). The anastomotic leakage rate was significantly higher in first group (17% vs. 8.5%, p< 0.05), while patient-reported HRQOL outcomes were similar between groups after the 12 month follow-up period.

**Conclusions:**

By using a modified Wallace technique, we were able to significantly lower anastomotic stricture and anastomotic leakage rates, which are major issues in minimizing both short- and long-term postoperative complications.

## INTRODUCTION

Radical cystectomy (RC) is the standard management of non-metastatic invasive bladder cancer (BCa), and is curative in the majority of patients with localized disease. Despite the popularity of continent urinary diversion and neobladder reconstruction, radical cystectomy with ileal conduit urinary diversion remains the most common curative surgical approach for patients with invasive bladder cancer (1). In regard to anastomotic technique, two Wallace surgical techniques have been described: medial wall (Wallace 1) or head-to-tail (Wallace 2) anastomosis. However, both techniques are associated with risk of stricture (bilateral ureteral obstruction) at the site of anastomosis (2). Ureteroileal anastomotic stricture (UAS) is an infrequent but potentially severe complication that may ultimately lead to renal impairment. In addition to patient- and disease-related risk factors, UAS can be a consequence of a poor surgical technique (3). Therefore, new techniques of ureteroileal anastomosis should be developed to reduce postoperative morbidity. For the primary end point, we hypothesized that the rate of ureteroenteric strictures would be at least 20% lower in the second group. Secondary end points included rate of anastomotic leak, surgical time, deterioration of the upper tract, intraoperative blood loss, rates of positive surgical margins (PSM), and patient-reported HRQOL outcomes 12-months post-operative. The objective of this study was to describe a modified Wallace I anastomosis surgical technique, and to compare perioperative outcomes, complications and anastomotic stricture rate in a contemporary series of patients who underwent open RC with modified Wallace anastomotic technique versus traditional ileal conduit.

## MATERIAL AND METHODS

The surgical protocol was approved by the University of Montenegro institutional review board and registered with the Ethical comitee of Clinical centre of Montenegro (Nr. 03/01-517-1) and conducted in accordance with the principles of the Declaration of Helsinki of World Medical Association. All patients provided written consent prior to enrollment in surgery. Post-operative patients were followed for a minimum of 12 months to provide complications and health related quality of life (HRQOL) data. The European Organization for the Research and Treatment of Cancer (EORTC) Quality-of-Life Core Questionnaire version 3 was used to measure HRQOL (4–6). Questionnaires were self-administered by the patients and completed before surgery and at the 12-month follow-up visit.

BCa patients scheduled for definitive treatment by open RC plus pelvic lymph node dissection (PLND) and ileal conduit urinary diversion were recruited from the urology clinic at Clinical Centre of Montenegro between January 2010 and January 2016. Eligible patients were aged ≥30 years and had BCa clinical stage T2-T3/N0-3/M0. Patients were excluded if they had previous pelvic radiation, clinical stage T4 or M1, or extensive prior abdominal surgery. Patients who were lost or died during follow-up were excluded from the final analysis. Postoperatively, all patients were placed on the identical treatment pathway and were followed every 3-6 months with routine history and physical exams, diagnostic imaging of the chest/abdomen/pelvis, urine cytology, and complete blood work (7).

Patients were randomized to be treated with one of two surgical techniques. Group I consisted of 70 patients treated with the Wallace 1 technique, where ureteral medial walls were anastomosed together and the free edges of the newly constructed ureteral plate were anastomosed to the proximal end of an open bowel segment (ileum). Group II consisted of 70 patients treated with a modified Wallace 1 technique. The modified Wallace 1 technique involved eversion of the ureteral plate and bowel mucosa edges, which were anastomosed together in a running fashion, while the outer anastomotic wall was augmented with sero-serosal interrupted sutures and finally retroperitonealized. In both groups, men underwent removal of the prostate and women underwent hysterectomy and bilateral salpingo-oophorectomy, if these organs were present. The extent of the PLND was left to the discretion of the surgeon, based on clinician preference and judgment and at a minimum, hypogastric, obturator and external iliac lymph nodes were removed bilaterally (7). In the second group, lymph node dissection templates were standardised including obturator, external/internal/common iliac, and presacral nodes (8). Finally, in case of positive lymph nodes, extended dissections removed the lymph nodes overlying the aortic bifurcation and continued to the takeoff of the inferior mesenteric artery.

## SURGICAL TECHNIQUE

**Figure 1:**
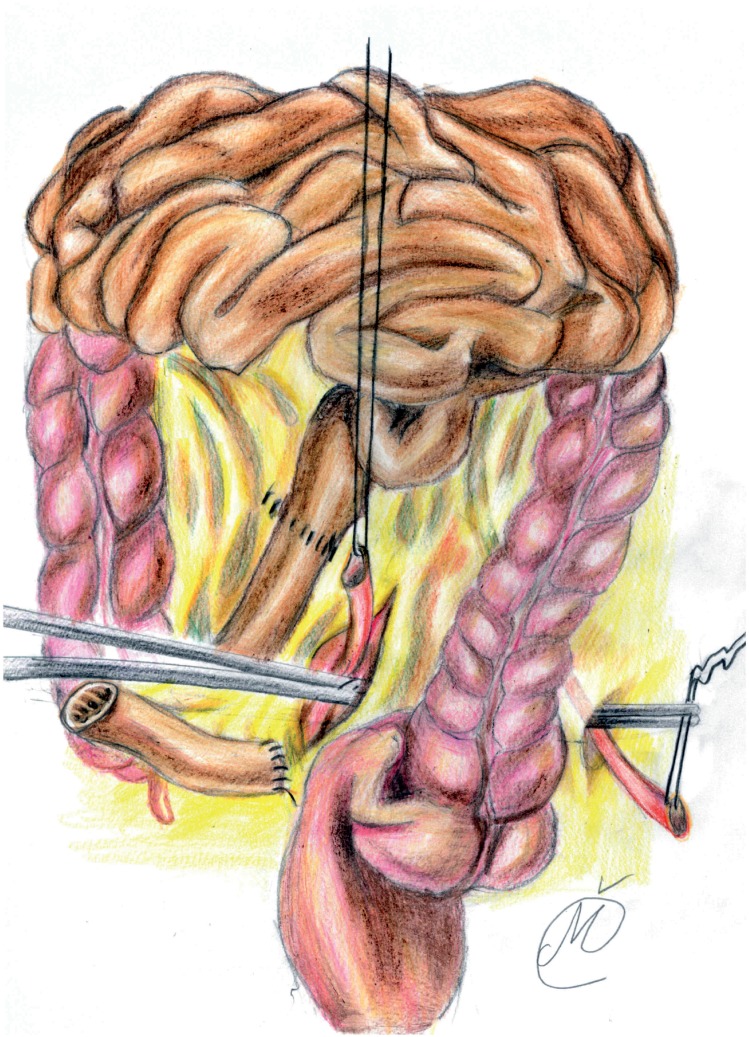
The mesentery window was closed with interrupted sutures, next, ureters were conjoined, with the left ureter transposed to the right side of the pelvis through a tunnel prepared at the base of the sigmoid mesentery in front of the common iliac vessels.

All surgical procedures were performed by a single expert, high-volume surgeon (P.K), who had a decade of experience in RC with Wallace 1 ileal conduit before the start of the study. After completing RC, extended pelvic lymph node dissection was performed. After identification of the ileocecal valve and distal ileum, a 10-15cm long ileal segment was isolated, approximately 20cm proximal. A long and straight incision of the mesentery, on both ends, was made using the Harmonic Focus long shears (Ethicon Endo-Surgery Inc. Cincinnati, OH, USA). A side-to-side ileo-ileal anastomosis was performed using a PDS 4-0 continuous suture. The mesentery window was closed with interrupted sutures and the isolated segment was flushed with saline and povidone iodine. Next, ureters were conjoined, with the left ureter transposed to the right side of the pelvis through a tunnel prepared at the base of the sigmoid mesentery in front of the common iliac vessels (9) (Figure-1). The redundant tract of both ureters was resected in order to obtain a tension-free ureteroileal anastomosis without angulation (3) and to improve vascular supply of the distal ureteral plate. Both ureters were spatulated for 2cm and laid adjacent to each other; the apex of one ureter was sutured to the apex of the other ureter with 4-0 Vicryl or polydioxanone (PDS) sutures (10).

**Figure 2:**
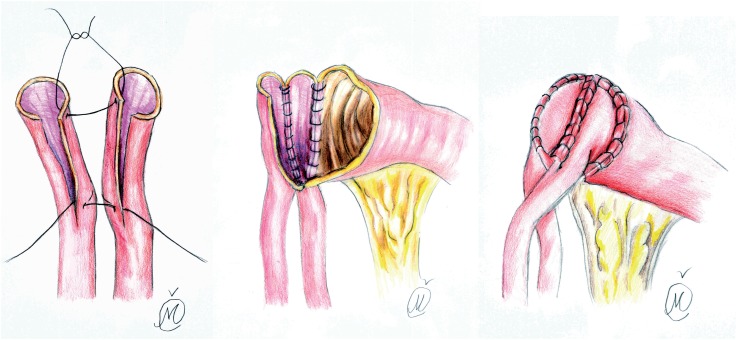
Ureters were spatulated and sutured together with continuous 4-0 Vicryl suture, while the lateral edges of the newly conjoined ureters were anastomosed to the proximal end of an open ileal conduit segment, using 4-0 PDS interrupted suture, according to the standard Wallace I technique.

In group I, the posterior medial walls of spatulated ureters were sutured together with continuous 4-0 Vicryl suture (the knots tied to the outside), over a 6 ch ureteric catheter, while the lateral edges of the newly conjoined ureters were anastomosed to the proximal end of an open ileal conduit segment, using 4-0 PDS interrupted suture, according to the standard Wallace I technique (1, 11) (Figure-2).

**Figure 3:**
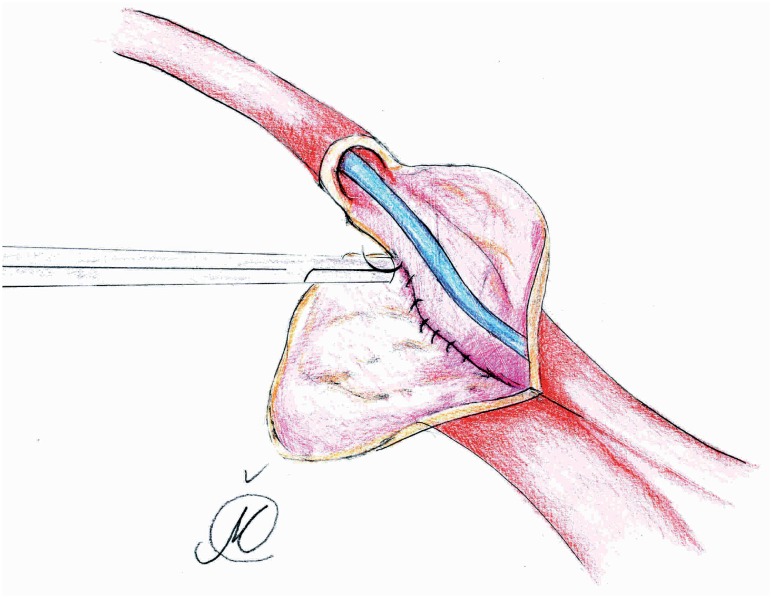
After each ureter spatulation, initial 5-0 PDS suture was placed at the apex of both ureters through all layers with muco-mucosal running suture of everted posterior medial ureteral wall edges (4-0 Vicryl), over a 6 ch or 8 ch ureteric catheter.

**Figure 4:**
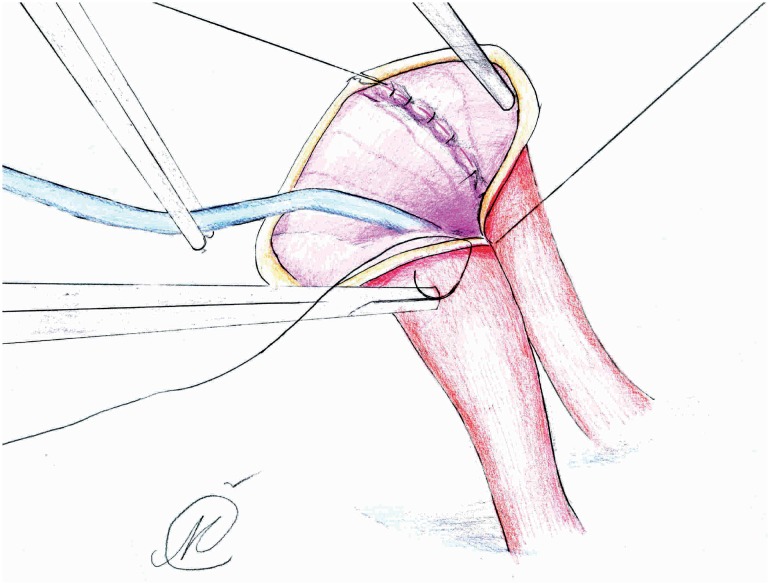
The needle reverses posteriorly to facilitates further muco-mucosal running suture of postero-medial ureteral wall edges (4-0 Vicryl), while several anterior wall sutures complete ureteral plate anastomosis.

**Figure 5:**
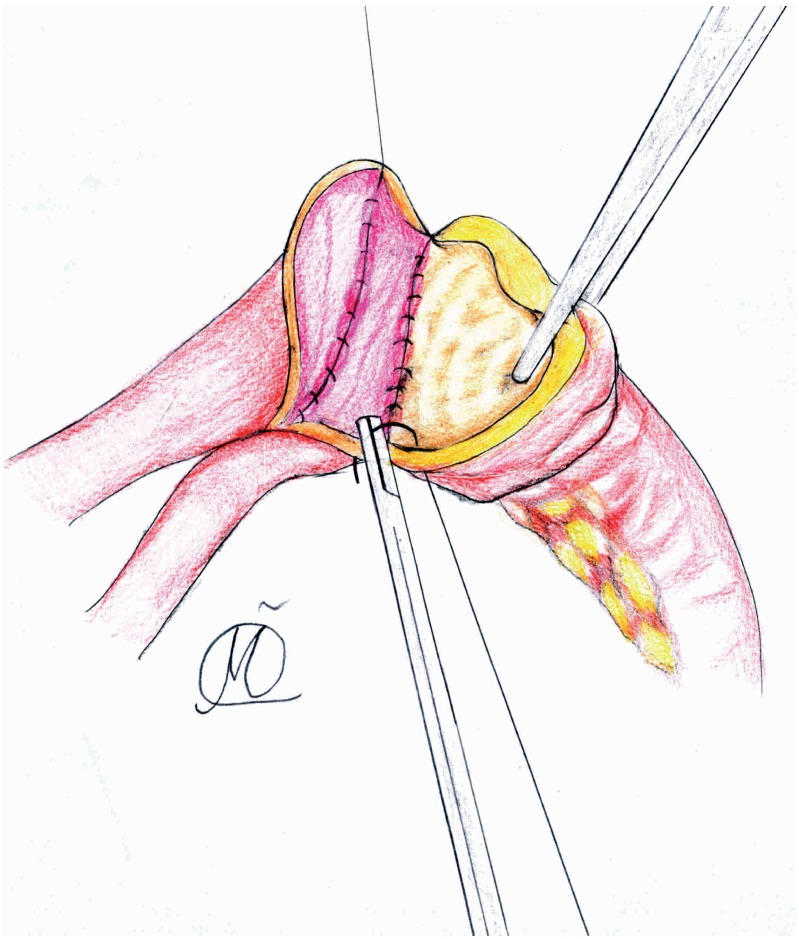
Lateral edges of the newly formed ureteral plate and the everted ileal mucosa (from the proximal end of conduit segment) were anastomosed in a running fashion.

**Figure 6:**
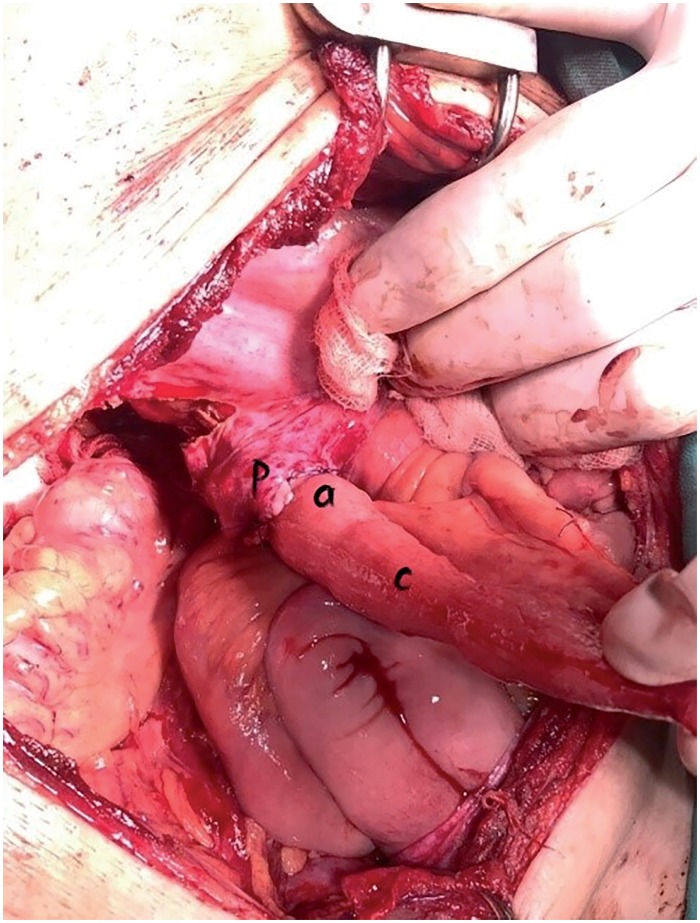
Ureteroenteric anastomosis with retroperitone-alisation of anastomotic line: a) peritoneal flap; b) ureteroenteric anastomotic site; c) conduit segment.

In group II, the isolated ileal segment was 20cm long, while each ureter was spatulated for 2.5-3cm and initial 5-0 PDS suture was placed at the apex of both ureters through all layers. Next, the needle reverses posteriorly to facilitates further muco-mucosal running suture of everted posterior medial ureteral wall edges (4-0 Vicryl), over a 6 ch or 8 ch ureteric catheter (Figures 3 and 4). Lateral edges of the newly formed ureteral plate and the everted ileal mucosa (from the proximal end of conduit segment) were anastomosed in a running fashion (Figure-5), while the outher anastomotic wall was augmented with sero-serosal interrupted suture (Vicryl 4-0 or PDS 4-0). At the end, conduit was retroperitonealized with the ureterointestinal anastomosis being placed in the retroperitoneum (Figure-6). This was accomplished by suturing the serosa of the conduit to the posterior peritoneum, above the anastomosis. Finally, in both groups, an abdominal stoma in the right iliac fossa was performed. The distal end of the ileal segment was first anchored to the rectus fascia with interrupted 4-0 Vicryl sutures and then to the skin, while an 18 Ch Folley catheter was placed in the conduit to allow for postoperative flushing (3). A Jackson Pratt drain was placed in the retroperitoneum a few cm away from the anastomosis. Ureteric catheters were sequentially removed at 7 and 8 days if the ileus resolved, while the Jackson-Pratt drain was removed one day later, after checking the drain creatinine level (12). Following local protocol, loopogram studies were performed at 3, 6, and 12 months, and then yearly, to assess for ureteroenteric anastomotic strictures (8).

Power calculations and statistical analysis Statistical analysis was performed with SPPS v16.0 (SPPS, Chicago, IL, USA). Blood loss, operative time, and time to discharge (hospital stay) were assessed as continuous variables and tested for normalcy using the Kolmogorov test. The Student T test and Mann Whitney U test were used to determine statistical significance. Dichotomous variables were compared using the Fisher's exact tests. Spearman correlation analysis was used to determine the correlation between age and baseline QoL scores. The difference between obtained values was considered significant when p <0.05, and highly significant when p <0.01. We also present descriptive statistics such as mean (SD) values, percentages and interquartile range (IQR), generated with SPSS.

## RESULTS

Our study enrolled 180 patients, of whom 140 were randomized and underwent RC/PLND; seventy were randomized to group I and seventy to the group II. Post-randomization distributions of group demographics, disease characteristics, and pathologic staging were not significantly different (Table-1). The mean (SD) follow-up time was 26.1 (5.7) months in group I and 25.2 (4.8) months in group II. Overall, there were 12 patients who experienced local recurrences (8.5%), and a total of 6 deaths were observed, 3 of which from BCa. These were excluded from the study. Neoadjuvant chemotherapy was given to 26 (37.14%) patients treated with standard technique and to 23 (32.8%) patients treated with the modified Wallace technique. PLND was performed in 60 patients (85.7%) in group I and in 63 patients (90%) in the group II (Table-1). There was no difference in the lymph node yield based on the extent of dissection between groups (4.3% vs. 4.9%). Mean blood loss was 810±250mL and 780±320mL in first and second groups, respectively.

**Table 1:** Patient characteristics and comparison of perioperative outcomes between groups.

Mean (SD)/Percentage (%)			
Demographic & pathological characteristics		Wallace I; (n=70)	Modified Wallace I; (n=70)
Age (years)		64 (17.26)	61 (14.12)
BMI, kg/m^2^, mean (SD)		22.5 (3.2)	23.8 (2.6)
Follow-up time, mean (SD)		26.1 (5.7)	25.2 (4.8)
Male, n (%)		34 (48.5)	38 (54.2)
Female, n (%)		36 (51.5)	32 (45.8)
**ASA score, n (%)**			
	2	21 (30)	24 (34.2)
	≥3	49 (70)	46 (65.8)
**Pathologic stage, n (%)**			
	T2	25 (35.7)	31 (44.28)
	T3	45 (64.3)	39 (55.72)
	Lymph node-positive patients, n (%)	43 (61.4)*	30 (42.8)
	Operative time (min.), SD	260 (25.31)*	330 (32.1)
	Estimated blood loss (mL), SD	810 (250)	780 (320)
	Hospital stay (days), SD	21 (4.6)	19 (3.4)
	Transfusion rate, n (%)	10 (14.2)	12 (17.1)
**Postoperative complications, n (%)**			
	Paralitic ileus	11 (13.8)	17 (20.2)
	Renal insufficiency	5 (7.14)	2 (3)
	Pneumonia	11 (15.7)*	21 (30)
	Pyelonephritis	27 (38.5)	12 (17.1)*
	Anastomotic leakage rate, n (%)	12 (17)	6 (8.5)*
	Anastomotic stricture rate, n (%)	8 (12)	2 (3)*
	Stricture treatment, n (%)	8 (100)	2 (100)*
	Antegrade DJ stent placement	4 (77.5)	1 (50)
	Percutaneus nephrostomy (PCN)	1 (10)	1 (50)
	Uretero-Intestinal reimplantation (UIR)	3 (12.5)	0*
	Relapse on follow-up, n (%)	7 (10)	5 (7.1)

* statistically significant difference between two groups (p <0.05)

During the follow-up period, anastomotic stricture was observed in 8 patients (12%) from the first and 2 patients (3%) from the second group (95% confidence interval for difference, p <0.05) (Table-1). The anastomotic stricture was diagnosed after new onset of hydrouretero-nephrosis (HUN) or after an increase in the preexisting HUN was visualized by CT scan and confirmed by loopogram (13). Four patients from group I and one patient from group II underwent antegrade DJ stent placement, one patient from group I and the patient from group II received percutaneous nephrostomy tube (PCN) as a definitive treatment. The remaining 3 patients (all in group I) underwent uretero-intestinal reimplantation. None of the patients with UAS were managed conservatively. Additionally, two patients from the group I (2.8%) developed left ureteral stenosis, proximal to the anastomotic site, both were managed conservatively. No patients from group II developed left ureteral stenosis. Surgical margin positivity did not differ significantly between groups (5.7% vs. 4.2%). The anastomotic leakage rate was significantly higher in first group (17% vs. 8.5%, p=0.03), as well as lymph node positivity (61.4% vs. 42.8%, p=0.04). Paralitic ileus was the most common early complication in both groups (14% vs. 20%), followed by pyelonephritis and pneumonia (Table-1). Patient-reported HRQOL outcomes were similar between groups after the 12 month follow-up period (Table-2).

**Table 2:** EORTC QLQ-C30 scores preoperatively and at 12 months follow-up in the Wallace group (n=70) and the modified Wallace group (n=70).

Mean (SD) EORTC QLQ - C30 score (modified)					
		Preoperatively		12 - month follow-up	
		Group I	Group II	Group I	Group II
		(Wallace)	(Modified Wallace)	(Wallace)	(Modified Wallace)
**Function scale**					
	Physical	71.3 (13.7)	73.5 (14.3)	74.6 (11.7)	77.9 (12.1)
	Cognitive	69.1 (20.6)	81.5 (15.8)*	72.1 (14.6)	75.7 (13.8)
	Emotional	68.3 (14.6)	72.7 (13.5)	56.3 (14.6)	68.2 (14.5)*
	Social	71.3 (9.7)	77.6 (12.8)	60.5 (10.5)	75 (13.8)*
	Global health status -QoL	46.3 (12.6)	50.2 (18.5)	48.7 (13.9)	52.6 (16.5)
**Symptom scale**					
	Dyspnea	15.3 (12.7)	12.6 (11.3)	17.3 (10.7)	15.8 (11.2)
	Pain	31.3 (14.2)	33.1 (12.7)	24.3 (15.2)	23.4 (11.7)
	Nausea/Vomiting	9.5 (5.4)	7.3 (6.6)	9.1 (10.2)	7.7 (8.6)
	Constipation	18.9 (17.7)	21.7 (13.4)	19.2 (18.7)	15.6 (11.4)
	Diarrhoea	9.8 (4.1)	12.5 (7.2)	13.7 (9.3)	10.1 (7.3)
	Financial difficulty scale	57.9 (29.5)	39.2 (35)*	66.2 (24.3)	60.2 (21)

* statistically significant difference between two groups (p <0.05)

During the follow-up period, mild metabolic acidosis was observed in 3 patients (4.2%) from the first and 6 patients (8.5%) from the second group and effectively treated with alkalinizing agents. The presence of more than a mild acidosis, which prompt an evaluation for obstruction or redundancy of the conduit, was not observed in second group, despite longer ileal segment.

## DISCUSSION

UAS is a well-documented complication after RC which can result in irreversible damage to the corresponding renal unit; the associated surgical revision also carries additional risk of morbidity (3). The long-term incidence of UAS after ileal conduit diversion ranges widely, between 2-22% (14–17). While the exact mechanisms of benign stricture formation are not known, it is thought to be predominantly due to ischaemia and subsequent scarring at the anastomosis (18). Although poor surgical technique and extensive ureteral mobilization could be major risk factors for UAS, it is evident that other factors may jeopardize the blood supply to the distal tract of the ureter, increasing the risk of stricture (3). Short ureteral spatulation, high tension on ureteroileal anastomosis, or short conduit segment are well known factors. Nevertheless, the type of anastomotic suture on ureteral plate and ureteroileal anastomosis may play significant roles in reducing UAS rate. According to our results, muco-mucosal running suture of conduit anastomosis augmented with interrupted sero-serosal suture, paired with longer ileal segment and longer ureteral spatulation, leads to a significantly lower rate of uretero-ileal stricture as compared to the standard Wallace I technique (3% vs. 12%, p=0.02). These findings bolster the assertion that meticulous handling, preparation and fine suturing technique of distal ureter are essential to minimize the risk of postoperative strictures and urinary leak (19). Indeed, our study also found a significantly lower rate of anastomotic leak among patients treated with modified Wallace I technique (8.5% vs. 17%, p=0.04). A study carried out by Katherine AA et al. (20) showed that the associated factors with increased risk of benign UAS include higher body mass index (BMI), ASA score >2, lymph node involvement, male sex, and a history of previous abdominal surgery. In our study, 100% of patients with UAS showed evidence of lymph node involvement and anastomotic leak, while ASA score >2 was determined in 80% of patients, suggesting that these variables may have predictive value on UAS.

Although anastomotic leak is one of the most challenging adverse events, occurring in up to 7% of cases (21), this complication could be prevented with adequate surgical technique, such as low tension at the anastomosis, long ureteral spatulation, and proper suturing technique (14). Muco-mucosal anastomotic pattern could reduce the anastomotic tension and occurrence of ureteral devascularization. Additionally, sero-serosal interrupted suture may improve the watertight of anastomotic line. All these factors, together with longer ureteral spatulation, proper length of ileal segment, ureteric catheter placement and excision of redundant ureteral tracts, could significantly lower the incidence rate of UAS and anastomotic leak.

Nevertheless, the rate of anastomotic leak is still high in our series compared to rates reported in current literature (14, 15, 22), indicating that meticulous surgical technique needs to be improved. However, this remains controversial (10), as early studies showed that everting anastomosis significantly increases the chances for severe adhesions (23). The findings we report support the opposite conclusion. In addition, Chen et al. (24) claim that continuous suture has no advantage over interrupted in bowel anastomosis, and neither does two layer (as compared to single layer) anastomosis. Other groups have reported that sero-serosal interrupted suture between ureter and ileum avoids the incidence of stenosis and preserves the upper tract (25), our findings align with that conclusion. Despite attention to surgical technique, devascularisation can still occur. Introduction of Intravenous Indocyanine green (ICG) may facilitate assessment of distal ureteric vascularity in patients undergoing uretero-enteric anastomosis and may reduce the risk of uretero-enteric anastomotic stricture following surgery (18).

According to contemporary literature (26), excluding UAS, the three most common complications associated with ileal conduit diversion are pyelonephritis (5-23%), urinary calculi (3-16%) and stomal complications. In our study, the incidence of postoperative complications varied between treatment groups. The most common postoperative complication was pyelonephritis (38% vs. 17.1%), followed by pneumonia (15.7% vs. 30%), paralitic ileus and anastomotic leak. The high incidence of pyelonephritis among patients treated with standard Wallace I technique is likely associated with the high rate of anastomotic leak (17%), which will certainly lead to renal impairment over time. While it is early to discuss renal deterioration two years post-operative, 7.5% of patients from the first group have already developed renal insufficiency. However, members of group II (who were treated with modified Wallace I technique) had a significantly lower incidence of pyelonephritis (17.1%) and a lower (though not significantly) occurrence of renal insufficiency (3%). This indicates the importance and effectiveness of our technique in reducing postoperative complication rates and precluding renal impairment. However, a longer follow-up period would provide stronger evidence of the impact of our technique on the incidence of renal impairment after RC.

In the present study, the baseline characteristics of the patients and QoL were measured immediately and 12 months postoperatively. Our treatment groups were similar with regard to the majority of baseline characteristics, lymph node positivity being an important exception. It is commonly believed that there are differences in the QoL outcomes of various diversions, but there are no published studies that have conclusively documented the superiority of one technique in terms of QoL (27, 28). Our results, however, demonstrate that patient self-rated emotional and social functional were significantly lower postoperatively among those in group I (56.3 vs. 68.2, 60.5 vs. 75). On the other hand, patients from group II remained in the same functional range as before surgery. This result is associated with lower incidence of UAS, urinary leakage, and pyelonephritis. This is additionally associated with less invasive treatment required, with regard to stricture.

The limitations of this study are the small size of randomization groups and the short followup period. In addition, the unusually high rate of anastomotic leak in patients within the first group could lead to research bias regarding effectiveness of modified Wallace technique, although the postoperative leakage rate was significantly reduced compared to first group. Nevertheless, single surgeon experience could be the major reason for this bias, which could be addressed by involving other highly trained surgeons. Finally, our quality of life questionnaire is simplified and not bladder cancer-specific, therefore urinary diversion-specific problems (urostomy problems, sexual functioning, etc.) were not assessed. Had we been able to use a bladder cancer-specific instrument, we could have more easily identified the causes of differences in QoL scores between the groups (6).

## CONCLUSIONS

By using a modified Wallace technique, we were able to significantly lower anastomotic stricture and anastomotic leakage rates, which are major issues in minimizing both short- and long-term postoperative complications. The present study provides important information about differences in QoL domains between patients undergoing standard Wallace I versus modified surgical technique, and the probable reasons behind these observed differences. Finally, additional studies with a longer followup period are necessary, as the endpoint of this study was too early to capture the majority of benign ureteroenteric strictures.
